# UKCTOCS update: applying insights of delayed effects in cancer screening trials to the long-term follow-up mortality analysis

**DOI:** 10.1186/s13063-021-05125-8

**Published:** 2021-03-01

**Authors:** Matthew Burnell, Aleksandra Gentry-Maharaj, Steven J. Skates, Andy Ryan, Chloe Karpinskyj, Jatinderpal Kalsi, Sophia Apostolidou, Naveena Singh, Anne Dawnay, Robert Woolas, Lesley Fallowfield, Stuart Campbell, Alistair McGuire, Ian J. Jacobs, Mahesh Parmar, Usha Menon

**Affiliations:** 1 MRC CTU at UCL, Institute of Clinical Trials and Methodology, University College London, 90 High Holborn, 2nd Floor, London, WC1V 6LJ UK; 2 MGH Biostatistics, Massachusetts General Hospital and Harvard Medical School, 55 Fruit Street, Boston, MA 02114 USA; 3 Department of Women’s Cancer, Institute for Women’s Health, University College London, 84-86 Chenies Mews, London, WC1E 6HU UK; 4 Department of Pathology, Barts Health National Health Service Trust, The Royal Hospital, Whitechapel Rd, London, E1 1BB UK; 5 Department of Clinical Biochemistry, Barts Health National Health Service Trust, Barts Health, 4th floor, Pathology and Pharmacy, 80 Newark St, London, E1 2ES UK; 6 Department of Gynaecological Oncology, Queen Alexandra Hospital, Cosham, Portsmouth, Hampshire PO6 3LY UK; 7 Sussex Health Outcomes Research and Education in Cancer, Brighton and Sussex Medical School, University of Sussex, Science Park Road, Falmer, Brighton, BN1 9RX UK; 8 Create Health, 150 Cheapside, London, EC2V 6ET UK; 9 Department of Social Policy, London School of Economics, Houghton Street, London, WC2A 2AE UK; 10 University of New South Wales, Sydney, NSW 2052 Australia

**Keywords:** UKCTOCS, Follow-up, Mortality analysis, Ovarian cancer, Cancer screening, Delayed effect

## Abstract

**Background:**

During trials that span decades, new evidence including progress in statistical methodology, may require revision of original assumptions. An example is the continued use of a constant-effect approach to analyse the mortality reduction which is often delayed in cancer-screening trials. The latter led us to re-examine our approach for the upcoming primary mortality analysis (2020) of long-term follow-up of the United Kingdom Collaborative Trial of Ovarian Cancer Screening (LTFU UKCTOCS), having initially (2014) used the proportional hazards (PH) Cox model.

**Methods:**

We wrote to 12 experts in statistics/epidemiology/screening trials, setting out current evidence, the importance of pre-specification, our previous mortality analysis (2014) and three possible choices for the follow-up analysis (2020) of the mortality outcome: (A) all data (2001–2020) using the Cox model (2014), (B) new data (2015–2020) only and (C) all data (2001–2020) using a test that allows for delayed effects.

**Results:**

Of 11 respondents, eight supported changing the 2014 approach to allow for a potential delayed effect (option C), suggesting various tests while three favoured retaining the Cox model (option A). Consequently, we opted for the Versatile test introduced in 2016 which maintains good power for early, constant or delayed effects. We retained the Royston-Parmar model to estimate absolute differences in disease-specific mortality at 5, 10, 15 and 18 years.

**Conclusions:**

The decision to alter the follow-up analysis for the primary outcome on the basis of new evidence and using new statistical methodology for long-term follow-up is novel and has implications beyond UKCTOCS. There is an urgent need for consensus building on how best to design, test, estimate and report mortality outcomes from long-term randomised cancer screening trials.

**Trial registration:**

ISRCTN22488978. Registered on 6 April 2000.

**Supplementary Information:**

The online version contains supplementary material available at 10.1186/s13063-021-05125-8.

## Background

Randomised controlled trials (RCT) are the cornerstone of the evidence base for clinical management of millions of patients across the world. RCTs evaluating the mortality impact of cancer screening typically involve large numbers of participants followed up over many years, sometimes decades. The general rule in clinical trials is strict adherence to the statistical analysis plan specified prior to unblinding and analysis of outcome data. Sometimes, during continued long-term follow-up of these trials, new understanding based on evidence from other trials and new analytical methods may require re-evaluation of the analysis plan.

One important example is the accumulating evidence in cancer-screening trials of a delay of several years before a mortality reduction is observed between the screen and control arms [1–3]. Almost all the cancer-screening trials, breast [4–14], prostate, colorectal, and lung [15–31] in their graphic representation of disease-specific mortality over time have reported a delayed difference (if present) between screen and control arms (Table 1). Most have an initial time window in the first several years after start of screening during which there is little or no mortality reduction, followed by one in which the reduction becomes evident [2]. These findings are in keeping with our understanding of how screening works. It reduces deaths by detecting cancers early, before they reach an incurable state. It is less likely to prevent cancer deaths occurring in the early years post randomisation as there is little chance to detect these cancers sufficiently early in their natural history. However, almost none of these cancer–screening trials have used analytical methods which formally allow for a non-constant effect (non-proportional hazards). All have described the screening effect using relatively simple methods, usually a single Poisson-based rate ratio (RR) [4, 12, 24, 30, 34, 35] or Cox model with a single hazard ratio (HR) estimate [18, 22]. A single HR is only appropriate if the reduction in hazard rates is relatively immediate and constant over time. In screening trials, such estimates cannot reliably describe the changing effects of screening on mortality over time.

**Table 1:** Summary of mortality analyses of randomised controlled cancer-screening trials

Trial name	Disease area	Country	Number of participants	Recruitment period	Number of screens	Screening period	Censorship date	Median FU from randomisation	Original analysis		LTFU analysis		No of years from randomisation to mortality reduction*
									Statistical analysis methodology	Final mortality reduction (95%CI)	Statistical analysis methodology (if different)	Final mortality reduction (95%CI)	
Two county	Breast	Sweden	162,981	1977	4	1977–1984	end 1984	5.93 years (mean) (29 years? LTFU)	“Mantel-Haenszel” techniques - stratified by county and age	RR = 0.69 (no CI reported; *p* = 0.013)	Negative binomial regression, robust SEs for cluster randomisation	RR = 0.69 (95% CI 0.56–0.84; *p* = 0.0001)	~ 4 years (Figure 1) [14]
Malmo	Breast	Sweden	42,283	1976–1978	5	1976–1986	end 1987	8.8 years (mean)	Relative risk (RR), test based CI	RR = 1.29 (95% CI 0.74–2.25)			No screening effect (no figure in analysis time) [32]
Gothenburg	Breast	Sweden	51,611	1982–1984	4–5	1982–1991	end 1996	11.8 years (mean) (~ 14 years LTFU)	RR, Poisson regression. Test based on Likelihood ratio	RR = 0.56 (95% CI 0.31–0.99; *p* = 0.046)	RR, Poisson regression adjusted for birth cohort	RR = 0.79 (95% CI 0.58–1.08; *p* = 0.14)	~ 0 years (Figure 1) [5]
Edinburgh	Breast	UK	54,654	1978–1985	2–4 (depending on cohort)	1978–1988	1992	~ 9 years? 10 years max (12.8 years (mean) LTFU)	Logistic regression modified for cluster randomisation and stratified by age. ITT	RR = 0.82 (95% CI 0.61–1.11) [RR with logistic regression?]	Same	RR = 0.87 (95% CI 0.70–1.06)	~ 6 years (Figure 2) [33]
UK Age Trial	Breast	UK	160,921	1991–1997	7	1991–2004?	end 2004	10.7 years (17.7 years LTFU)	RRs, no detail. ITT	RR = 0.83 (95% CI 0.66–1.04; *p* = 0.11)	Poisson regression (presumably as before).	RR = 0.88 (95% CI 0.74–1.04; no *p*-value)	~ 3 years (Figure 2) [34]
ERSPC	Prostate	Europe (7 countries)	162,387 (in the core age group)	1991–2003	up to 3?	1991–2003	end 2006	9.0 years (13 years LTFU)	Poisson regression to estimate mortality ratio (RR), stratified by centre and age group. ITT	RR = 0.80 (95% CI 0.65–0.98; *p* = 0.04).	Same	RR = 0·79 (95% CI 0·69- 0·91; *p* = 0·001)	~ 7 years (Figure 2) [31]
SCORE	Colorectal	Italy	34,292	1995–1999	1	1995–1999?	2006?	11.4 years	RRs based on average mortality rates (Poisson distribution). ITT	RR = 0.78 (95% CI 0.56 to 1.08)			~ 5–6 years (Figure 2c) [35]
NORCCAP	Colorectal	Norway	98,792	1999–2001	1	1999–2001	end 2011	10.9 years (14.2 years LTFU (mean))	HRs from Cox model, adjusted for age. ITT	HR = 0.73 (95% CI, 0.56–0.94; *p* = 0.02)	Same, except primary analysis now has separate estimates for men and women	Men HR = 0.63 (95% CI 0.47-0.83) Women HR = 1.01 (95% CI 0.77-1.33)	~ 5–9 years (~ 3 years for men) (Figure 2c) [21]
PLCO	Prostate	USA	76,693	1993–2001	4–6	1993–2005?	2008	11.5 years (14.8 years LTFU)	RRs assuming Poisson distribution. ITT. No mention of WLR test and no *p* value given subsequently	RR = 1.13 (95% CI 0.75 to 1.70)	Same	RR = 1.04 (95% CI 0.87-1.24)	no screening effect (Figure 1) [26]
PLCO	Lung	USA	154,901	1993–2001	4	1993–2005?	end 2009	11.9 years	RRs assuming Poisson distribution. Adjusted p for sequential analyses (interim). No mention of how p calculated	RR = 0.99 (95% CI, 0.87–1.22; *p* = 0.48)			no screening effect (no figure) [20]
PLCO	Colorectal	USA	154,900	1993–2001	2	1993–2004	end 2009	11.9 years (15.8 years)	Weighted (0,1) LR test with RRs assuming Poisson distribution. Adjusted p for sequential analyses (interim)	RR = 0.74 (95% CI 0.63 to 0.87; *p* < 0.001)	Same for RRs though notably no test/p-value	RR = 0.75 (95% CI 0.66–0.85)	~ 3 years (Figure 2a) [36]
PLCO	Ovarian	USA	78,216	1993–2001	4–6	1993–2005?	28 Feb 2010	12.4 years (14.8 years LTFU)	Weighted (0,1) LR test (one-sided) with RRs assuming Poisson distribution. Adjusted p-value for sequential analyses (interim)	RR = 1.18 (95% CI, 0.82–1.71) CI sequentially adjusted. No *p* value reported possibly because test was 1-sided?	Same for RRs though notably no test/p-value (also added a Cox model)	RR = 1.04 (95% CI 0.87–1.24)	no screening effect (Figure 1) [37]
NLST	Lung	USA	53,454	2002–2004	3	2002–2007	end 2009	5.4 years (mean)	RRs assuming Poisson distribution. Adjusted p for sequential analyses. Weighted	RR = 0.80 (95% CI 0.73–0.93; *P* = 0.004)			~ 1.5 years (Figure 1B) [24]
UK Flexible Sigmoidoscopy Screening Trial (UKFSST)	Colorectal	UK	170 034	1994–1999	1	1994–1999	31 Dec 2014	17·1 years	HRs from Cox model. ITT	HR = 0.69 (95% CI 0.59–0.82; p<0.0001).	Same	HR = 0.59 (95% CI 0.49–0.70)	~ 3 years (Figure 1G) [17]
Canadian National Breast Screening Study (CNBSS)	Breast	Canada	50,430	1980–1985	5	1980–1985	end 1991	8.5 years (mean) (25 years LTFU)	*T* test on difference of proportions	RR = 1.36 (95% CI 0.84–2.21)	Cox PHs model	HR = 0.99 (95% CI 0.88-1.12; *p* = 0.87)	no screening effect (Figure 3) [11]

*Estimate of mortality curve separation comes from visual inspection of appropriate published mortality plot if provided. The Figure number and paper reference are given to allow the reader to make their own judgement

*FU* follow-up, *LTFU* long-term follow-up, *RR* rate ratio, *HR* hazard ratio, *ITT* intention to treat analysis, *LR* log-rank

Alongside, new analytical methods have been developed for trials lacking treatment proportionality. Tests that combine evidence from more than one aspect of the data have gained popularity as a way to mitigate the effects of potential but unknown non-proportionality of hazards, although some may work best in a specific scenario. The “joint test” appears in simulations to be preferentially beneficial under late effects [38, 39] whilst the “combined test” appears to be preferentially beneficial under early effects [40, 41]. Another recent addition is the Versatile test [42], which seeks to cover all bases by combining three (weighted) log-rank tests giving good power for the test under early effects, proportional hazards (PH) and late effects, respectively. These tests are likely better suited than the Cox model for analysis of outcomes which are non-proportional across the duration of a trial.

In the United Kingdom Collaborative Trial of Ovarian Cancer Screening (UKCTOCS) too, the initial mortality analysis in 2014 used a PH Cox model and reported an average mortality reduction estimate. However, given the growing external evidence, there have been extensive discussions within the UKCTOCS trial committees to ensure the outcome data is analysed appropriately. We believe that this issue will be important for any long-term cancer screening trial. The Cox model, while valid, could be viewed as restrictive and failing to utilise the most appropriate analytical approach, given the delayed mortality reductions seen in many screening trials across a range of cancers (Table 1) [14, 17, 24, 31]. Furthermore, retention of the Cox model based on pre-specification may result in suboptimal interpretation of UKCTOCS data and therefore an abrogation of our responsibility to the huge collective investment by the trial volunteers, the funding agencies, charities, the National Health Service (NHS), researchers and most importantly women who develop ovarian cancer in the future. This is balanced by a concern that changes to the 2014 analysis plan could be controversial and lead to criticism of cherry-picking methodology that gives the “best” test result.

Many trialists may face similar dilemmas, when new evidence suggests that trial design, conduct or analysis may need to be amended. Decisions are often made by the Trial Management Committee (TMC) with input from independent oversight bodies such as a Trial Steering (TSC) or Scientific Advisory (SAC) Committees. We report on the process we undertook in UKCTOCS to re-examine our approach for the upcoming analysis (2020) of the primary mortality outcome at the end of extended follow-up and how we addressed the issue of delayed effects.

## Methods

Between 2001 and 2005, 202,638 postmenopausal women aged 50–74 were recruited to UKCTOCS. They were randomised to screening using a longitudinal serum CA125 algorithm (multimodal group, MMS, 50,640), transvaginal ultrasound (ultrasound group, USS, 50,639) or no screening (control group, C, 101,279) as described previously [43–45]. Women in the screen groups underwent screening until the end of 2011 and received a median of nine annual screens. At median follow-up of 11.1 years (administrative censorship 31 Dec 2014), a higher proportion of women were diagnosed with low-volume (stages I, II and IIIa) tubo-ovarian cancer in the MMS (40%; *p* < 0.0001) compared to the C (26%) group. The Cox model indicated a trend to mortality reduction in favour of MMS (HR 0.85; 95% CI 0.70–1.03, *p* = 0.10) and USS (HR 0.89; 95% CI 0.73–1.07, *p* = 0.21), which was not statistically significant at the 5% level. A Royston-Parmar (RP) flexible parametric model showed that HR varied over time. In the MMS group, it was 0.92 (95% CI 0.69–1.20) in years 0–7 and 0.77 (95% CI 0.54–0.99) in years 7–14. In the USS group, it was 0.98 (95% CI 0.74–1.27) in years 0–7 and 0.79 (95% CI 0.58–1.02) in years 7–14 [43]. Follow-up was extended to 30 June 2020 to assess the long-term mortality impact (LTFU UKCTOCS) [43, 46]. Final receipt of death data from the registries is anticipated by the end of September 2020, with unblinding and analysis planned for November 2020.

To ensure independent input into our statistical conundrum, the TMC proposed seeking the views of a broad panel of international experts with statistical and screening trial expertise who had not been involved in any aspect of UKCTOCS. The process was developed through detailed discussions with the independent members of the TSC. In September 2019, 12 experts (Table 2) were approached by the Trial Statistician for advice. They were sent a letter briefly describing UKCTOCS together with a summary of the current evidence from other cancer-screening trials, importance of pre-specification and our 2014 mortality analysis results. Three potential options for the primary analysis of the extended follow-up data developed with the TSC were described sequentially, each including possible pros and cons, in a neutral manner. These were:

A) Analyse all outcome data (2001–2020) using the PH Cox model of the original UKCTOCS analysis, representing the pre-specification viewpoint.
B) Analyse only the outcomes that occurred since the original censorship (31 December 2014), either assuming PH or not, to address the view that data should not be re-used, without formal statistical accommodation for multiple analyses.
C) Model all outcome data using a method of analysis and model that allows for a late effect of screening on mortality and reflects current understanding of cancer-screening trials—a pragmatic evidential approach. The specific model suggested for (C) was the RP model [47] as it had been used as a secondary analysis method for the 2014 analysis [43].

**Table 2:** Summary of experts' choices and their additional suggestions if not in concordance with A, B or C

Expert	Expertise	Choice	Additional suggestions
EX1	Biostatistics, public health	A	Suggests only include cancers diagnosed from period of intervention.
EX2	Biostatistics, clinical trials and cancer research	A	
EX3	Statistics	A	Ticked “alternative” but suggested hybrid of A for testing and C for estimation—interpreted as A
EX4	Cancer epidemiology, prevention and screening	Change analysis	Suggested “number needed to screen”.
EX5	Biostatistics, cancer epidemiology	Change analysis	Did not complete form but indicated choice by email, test based on difference of restricted mean survival time (RMST).
EX6	Biostatistics and epidemiology	Change analysis	Suggested splitting data into yearly bins and assess HR in each, possibly with smoothing. Avoid single HR.
EX7	Biostatistics, clinical trials and cancer research	C	Did not complete form but indicated choice by email. Prefers more parsimonious model with interpretable parameters.
EX8	Biostatistics, clinical trials	C	
EX9	Biostatistics, public health	C	Prefers more parsimonious model with interpretable parameters.
EX10	Cancer epidemiology, public health	C	Also suggests “versatile weighted log-rank test”
EX11	Statistics, public policy	C	
EX12	Biostatistics	–	Did not respond within timeframe

Experts were asked to critique and state a preference or suggest another option (Supplementary Materials 1). Results were collated and summarised based on (1) indicated choice of A, B, C or other and (2) pertinent comments provided.

## Results

In total, 12 individuals were contacted from the UK (5), the USA (5), Canada (1) and Belgium (1) and 11 responded (see acknowledgement). Their anonymised responses can be found in Table 2 and Supplementary Table 1.

Eight (73%) of the 11 experts recommended changing the pre-specified analysis to one that more appropriately allows for a delayed effect (Table 2). *EX4* was not troubled by the shift from a pre hoc to post hoc decision—“reason” should have a role in science. Similarly, *EX8* argued “a conclusion should be reached based on a proper consideration of the full evidence” and use scientific principles—“full information from data should be extracted”. Indeed, rather than viewing it as “data-dredging” or “changing the endpoint”, *EX8* described this approach as just “using common sense”. *EX9* felt the lack of (complete) pre-specification a weakness, but not “a violation of good scientific principles”. For “a major and definitive screening trial ….. such regulatory constraints should not be the primary consideration” but instead “approximating the truth as well as possible”. *EX11* was not persuaded by the pre-specification argument and claimed keeping a plan that is less preferable “turns research rules into an irrational, mindless, and restricting obsession with methodological procedure”; “rules have a purpose, but when the higher priority is understanding phenomena in a reasoned disciplined way… then a compelling argument can be made to deviate from them”. *EX11* stated that no screening trial has shown an immediate effect and appealed to the common sense of the scientific audience; “we can discern the difference in attempts by a study team to game the analysis to gain statistical significance, from a good faith effort to apply a statistical technique that is more appropriate for the data”. Different screening trials will have different results and delayed effects, all dependent on differing facets of trial design and the cancer itself, the effects of which are largely unknown until we do the study. “Point is, we are still learning how to design and analyse RCT screening trial data.”

Three of the eleven (*EX2*, *EX3*, *EX1*) believed that we should retain the initial analysis approach (option A). This was based on the pre-specification argument—“avoids the appearance of trying to get a significant result by changing the test” (*EX2)*, “maintains credibility in the scientific community” (*EX3*), “most likely to be accepted as valid by the cancer research and policy community” (*EX1*). However, *EX1* did suggest modifying the pre-specified plan to limit analysis to only cancers diagnosed within the screening period.

Of the eight who suggested changing the pre-specified analysis, five (*EX7*, *EX8*, *EX9*, *EX10* and *EX11*) explicitly selected approach C (using all acquired outcome data and a model that allows for delayed effects). While there were positive comments about the suggested RP model (credibility due to pre-specification *EX7*, informative of the screening effect over time *EX9*), none gave a clear endorsement of this approach. The main reason was interpretability (*EX7*, *EX9*, *EX4*, *EX6*). *EX10* noted that power was little studied under various “flavours” of non-PHs, and suggested separating testing from estimation, opting for a versatile weighted log-rank test for the former. *EX4* and *EX6* formally indicated an alternative option. *EX6*’s preference was for dividing the data into yearly bins and estimating the HR in each, possibly with some smoothing. *EX6* argued extensively we should avoid a single HR estimate, which will provide “a very blurred, incomplete and misleading picture of how much/little good screening did for the 100,000 participants screened, or of how much future women might expect from a screening regimen based on these screening tools.” *EX4* stated that the number needed to screen was the most suitable measure for a screening study. *EX5* recommended a test based on the difference of restricted mean survival times (RMST) which “does not need any modelling and the results can be interpreted easily clinically”.

None of the 11 responders chose approach B. This was mainly because it did not use the full dataset. In addition, there were concerns that it could lead to “unfavourable early results” (important data) being censored (*EX11*) and a “disconnected” HR (*EX6*)*.*

Based on the feedback, we decided to change the primary analysis test for LTFU UKCTOCS. Table 3 summarises the major pros and cons of available approaches to dealing with non-PH in terms of tests. We used two main criteria to choose the specific test—(1) minimal a priori specification on the specific form of the mortality difference over time (2) able to accommodate delayed effects while maintaining good power in a variety of potential scenarios. Based on these criteria, we opted for the Versatile test [16], suggested by *EX10*. The RP model was retained to estimate absolute differences in disease-specific mortality at 5, 10, 15 and 18 (our estimate of the upper limit of reliable follow-up given administrative censorship on 30 June 2020) years. Options A and B were included as secondary analyses of the primary mortality outcome. These amendments were incorporated into the statistical analysis plan (20 February 2020), which was endorsed by the independent TSC.

**Table 3:** Summary of pros and cons of potential statistical tests that could be used when there is a time varying mortality difference (non-proportional hazards)

Method	Pros	Cons
Weighted log-rank test	Not model-based	Need to formally pre-specify the expected mortality differences over time (functional form of the HR) for the test to have statistical validity. This may prove difficult given that differences will depend on the natural history of the cancer, screening strategy, number of screens, years of follow-up, etc.
	Known to improve power in situations of non-PH.	There is an associated risk of mis-specifying the form of the HR, and simulations suggest incorrectly assuming a late effect, for example, may incur a greater penalty than assuming PHs under early or late effects [33, 47].
	Most widely used and established test for non-PHs in clinical trials	Subjects’ deaths are given a differential (and arbitrary) weighting which may be hard to justify. A further conceptual problem with weights based on the data is that if a trial subsequently reports again, the weight allocated to each event will change, likely significantly.
Flexible parametric model such as the Royston-Parmar (RP) model (cubic splines) or fractional polynomial (FP) survival model (joint test of all screen arm related terms)	No need to pre-specify specific functional form of the mortality effect	No precedence for use as primary analysis in RCTs
	Can mimic a non-PH function to almost arbitrary degree.	Flexibility makes it easy to over fit and include random data artefacts.
		Power properties not well known. Will lose power with too many model parameters.
	Allows one to accurately describe the hazards and their ratio over time.	Need to pre-specify number of knots/degrees of freedom and placement of knots for RP model. FP model requires choice of selection of powers and degree. Can be guided by information criteria but then data dependent, and may reflect artefacts.
	Relatively easy to fit	Test, as proposed, considers if mortality curves are “different”. Significant result could theoretically result from crossing curves, even curves with no difference in area under the curve.
Weibull model (with separate shape parameters for group)	Can reflect simple time-varying differences in mortality curves succinctly	Unlikely to capture more complex curves sufficiently. All hazard functions must be monotonic (constant decrease or increase)
	Easy to fit	
Cox model with time varying coefficient (TVC)	Extension of Cox model, so perhaps more readily acceptable given prior use	Need to pre-specify function of time that the non-PHs apply to—usually a simple linear or log function of time
	Able to incorporate non-PHs without specifying differences in mortality curves (functional form). For example, choose linear function of time, then time-varying effect could be linear decreasing or increasing.	Interpretation not straightforward
		Awkward and (very) time-consuming to fit (splits data at each failure) No definite agreement on test of significance. Could be similar to the joint test on 2 degrees of freedom.
	No need to consider baseline hazard function	
Difference in restricted mean survival time (RMST)	No need to be model-based, can use non-parametric estimation.	Need to pre-specify choice of time restriction, possibly including initial time *t*0, as well as final time limit *t*1.
	Can reflect any time-varying difference in mortality - estimate of RMST difference graphically corresponds to the difference in area between the respective survival curves.	
	Do not need to speculate on particular form of time varying difference in mortality. However choice of time restriction may depend on expectation of difference (HR functional form).	May be time consuming to estimate, including standard error.
	Gives a meaningful single summary estimate even with non-PHs	As the test looks for differences in area under the curve, survival curves that come back together can result in a significant test result.
Combined test (of Cox test with a permutation test based on RSMTs on 2 df)	Simulations suggest power not much lower than Cox alone under PHs and more powerful in more situations than joint test [33, 47].	Difficult to explain
		Time-consuming to fit (permutation test).
		Issues of RMST (see above)—choice of time restriction
	Enhanced power for early effect	Simulations suggest not powerful for late effects
Joint test (of Cox proportional screen arm effect + Grambsch-Thurneau non-PH test on 2 df)	Test based on results of the Cox model (screen arm effect and the Schoenfeld residuals), so perhaps more readily acceptable given prior use of the Cox model	Simulations suggest better under late effects but not good power for early effects [33, 47].
	Relatively simple test (with degree of intuitiveness), but more powerful than just screen arm effect under non-PHs	
Combination tests such as Versatile Test (maximum test statistic of 3 weighted tests—early, PHs, late effects) or “max-combo” (also includes “middle” effects)	Not model-based	Appears complicated (need for reference to a correlated multivariate z-distribution for test statistic)
	Provides good power in all situations, covers bases with small price in efficiency	Not the most powerful test.
	Best choice if one wants to be agnostic of specifying the time varying mortality difference	Can feasibly reject the null hypothesis both in favour of the study arm and of the control arm using the same data.

## Discussion

Given the now large body of evidence of a delay in mortality reduction in long-term cancer-screening randomised trials, and the majority view of independent statistical, epidemiological and screening trial experts, we altered the approach for our primary mortality analysis for the LTFU from that used for our 2014 analysis. The new approach allows for a delayed effect in contrast to our previous analysis which assumed a constant screening effect. There were a variety of opinions on the specific test which suggests an urgent need for consensus building on how best to design, analyse and report mortality outcomes in cancer-screening trials.

Our decision to change the statistical analysis plan for extended follow-up is a significant decision. The large majority of the published cancer-screening trials [17, 25, 26, 31, 33, 34] have retained the same primary mortality analysis methodology for both their initial and extended follow-up analysis (Table 1). The only exceptions we found were the Two County trial which used negative binomial regression [14] for follow-up analysis in place of Mantel-Haenszel stratified risk-ratios [12] and the Norwegian Colorectal Cancer Prevention Trial (NORCCAP) which changed the primary analysis from overall population to subgroups based on gender [21]. In the Two County trial, whilst no explanation was given, the change was not substantive; both initial and follow-up methods estimated risk ratios. For NORCCAP, “because substantial heterogeneity existed between women and men, the steering committee decided to present results for women and men separately”, which may be argued as a significant post hoc data-driven amendment. None of the trials as far as we are aware sought independent expert opinion. In contrast, we undertook an external consultation. Although the independent expert panel was not unanimous, the majority concluded that a rational argument for revision outweighs that of procedure and pre-specification, and recommended choosing the most appropriate test that allows for a delayed effect. We accepted the view of *EX7* that one should “do what you yourselves think is the most effective and secure analysis of all your data, bearing in mind the current state of information about the field.” There will be debate about our decision, which we welcome, given the broader implications.

A number of factors contribute to a delayed mortality effect. In the early trial-years, the absolute death rates are low as a result of eligibility criteria which exclude women with cancer diagnosis. The time interval for an individual to be diagnosed with cancer after joining the trial and then dying of the disease also contributes to the delay in separation of the mortality curves. Additionally, the impact of screening on cancers detected at the initial prevalence screen is reduced, as these are necessarily more advanced when screen-detected compared to screen-detected cancers in later years. The performance of most screening strategies improves over time as the number of screens accumulate and the teams involved become more experienced. This is magnified when longitudinal biomarker algorithms are used, as they are based on detecting change from baseline. Conversely, the length of follow-up after end of screening may reduce the mortality difference as follow-up nears conclusion, as the longer the interval, the greater the dilution of screen-detected cancers by cancers that develop after the end of screening [34].

The PLCO colorectal [29] and ovarian [19] trials used a test that has better power for the delayed effect described above. Both used the weighted log-rank test, which is perhaps the best known method for improving power in such situations. However, it requires correctly anticipating the specific form of the mortality difference over time, which will depend on the natural history of the cancer, screening strategy, number and frequency of screens and years of follow-up. We have chosen the Versatile test [42], introduced in 2016, which does not require pre-specification of the mortality difference over time. It combines three (weighted) log-rank tests appropriate for capturing early effects, PH and delayed effects, respectively. It is therefore versatile enough to maintain good power in all potential scenarios, rather than optimal in any given scenario.

Unlike other trials, including the PLCO colorectal [29] and ovarian [19] trials, who measured the screening effect using a single “averaged” rate-ratio, we will use a flexible parametric model to estimate absolute differences in disease-specific mortality at 5, 10, 15 and 18 years. This is in keeping with the growing view that to adequately describe what might be achieved with a particular cancer screening strategy, a more comprehensive set of time-specific measures needs to be reported. Hanley et al. has extensively re-analysed cancer screening trial data and shown that a one-number summary measure systematically dilutes the estimate of mortality reduction that results from screening [2]. In the most recent re-analysis involving breast cancer screening data from Funen, Denmark, the average mortality reduction was 18% using a PH model and ranged from 0 to 30% when a non-PH model was used that considered the impact at different points over time. The reductions were largest for periods where sufficient time had elapsed for the impact to manifest [48]. It is important to note that our estimates of screening efficacy will not necessarily capture the screening effect of a screening program, where participants would likely start screening at age 50 and continue for possibly 25 years. However, once results of our primary analysis are published, it will be possible for groups around the world to use our data to model effectiveness over a longer timeframe and in multiple settings.

The key strength of our approach is the independent and transparent process we have adopted to address a challenging issue and the criteria we used to choose a new specific approach. This involved accommodating delayed effects while maintaining good power in a variety of potential scenarios and requiring minimal a priori speculation on the specific form of the mortality difference over time. A limitation is that given the orthodoxy surrounding pre-specification for analysis of trials, we have retained the original Cox model with an averaged HR over time as an estimate for our secondary analysis.

The screening community is only beginning to understand the challenges posed by long-term cancer-screening trials. Mortality reductions may have been underestimated across cancer types by not considering their timing. Given the importance of early detection in many national cancer strategies, we hope our report will accelerate much needed consensus building on how best to design, analyse and report trials testing cancer screening strategies—as it is clear our currently accepted and widely used methods are insufficient. We also hope it will encourage debate and transparency on how advances in understanding and new analytical methods can be evaluated and incorporated into long-term trials.

## Supplementary Information

**Additional file 1: Supplementary Material 1.** Cover Letter to Independent International Expert panel, Outline of Options, Comment Form. **Supplementary Table S1.** Summary of Responses from Independent International Group.

## Data Availability

Table 2 and Supplementary Table 1 contain the exact comments provided by the experts.

## Abbreviations

UKCTOCS: United Kingdom Collaborative Trial of Ovarian Cancer Screening
LTFU UKCTOCS: Long-term follow-up of the United Kingdom Collaborative Trial of Ovarian Cancer Screening
RCT: Randomised controlled trial
RR: Rate ratio
HR: Hazard ratio
CI: Confidence interval
PH: Proportional hazards
TMC: Trial Management Committee
TSC: Trial Steering Committee
SAC: Scientific Advisory Committee
MMS: Multimodal group
USS: Ultrasound group
RP: Royston-Parmar model
NORCCAP: Norwegian Colorectal Cancer Prevention Trial
PLCO: Prostate, Lung, Colorectal and Ovarian Cancer Screening Trial
